# Limping as the first manifestation of malignant round cell tumor

**DOI:** 10.1186/1546-0096-12-S1-P281

**Published:** 2014-09-17

**Authors:** Leila Mahboobi, Mehrnoush Hassas Yeghaneh, Reza Shiari

**Affiliations:** 1 Pediatric Rheumatology, Shahid Beheshti University of Medical School, Tehran, Iran, Islamic Republic Of

## Introduction

A 3.5 -year old boy was referred Out-patient Clinic of Pediatric Rheumatology in Mofid Children’s Hospital because of limping. His problem was started 2 weeks before admission which accompanied by mid foot arthritis.

## Objectives

This case is a report of midfoot arthritis that is a unusual manifestation of malignancy in children. there is not any special method for this abstract.

## Methods

He was evaluated for the cause of limping that the results were as followings:

His Complete blood count showed normal levels of leukocyte without neutropenia, however, mild anemia (Hgb: 10.8 mg/dl) and thrombocytosis (578000).

PPD test was negative. Acute phase reactants like ESR and CRP was elavated.

## Results

Whole body bone scan revealed asymmetrical increased up take in ankle and linear increased up take in left metatarsus (3th).

His abdominal CT Scan showed multiple variable size hypodense lesions in bilateral kidneys and buldging and distortion of renal border.

Bone marrow biopsy performed that revealed positive for CD45 and CD3.

His renal lesions biopsy confirmed the diagnosis of Malignant Round Cell Tumor compatibale with T- Cell Leukemia and finally he went under the chemotherapy in oncology ward.

## Conclusion

As the result of this article we conclude that midfoot arthritis is an alarming sign in children because of the assosiation with malignacies. So it is important to exclude malignacies in children whom presenting with only midfoot arthritis.

## Disclosure of interest

None declared.

